# Caregiver perspectives and preferences for acute otitis media management

**DOI:** 10.1017/ash.2021.242

**Published:** 2021-12-22

**Authors:** Holly M. Frost, Amy Keith, Thresia Sebastian, Timothy C. Jenkins

**Affiliations:** 1 Department of Pediatrics, Denver Health and Hospital Authority, Denver, Colorado; 2 Office of Research, Denver Health and Hospital Authority, Denver, Colorado; 3 Department of Pediatrics, University of Colorado School of Medicine, Aurora, Colorado; 4 Division of Infectious Diseases, Department of Medicine, Denver Health and Hospital Authority, Denver, Colorado; 5 Division of Infectious Diseases, Department of Medicine, University of Colorado School of Medicine, Aurora, Colorado

## Abstract

Surveyed caregivers of children in Denver, Colorado, with acute otitis media (AOM) preferred immediate antibiotics over delayed antibiotics or observation. Overall, 77% stated that they would immediately fill a prescription written as delayed. In contrast, 86% of caregivers favored whichever duration was recommended by the provider or the shortest duration necessary.

Acute otitis media (AOM) is the most cited indication for antibiotics in children.^1^ Even though up to 81% of AOM episodes will resolve without antibiotic treatment, 95% of children with AOM are prescribed an antibiotic and most children 2 years and older are prescribed antibiotics for longer than recommended durations.^2,3^

Several strategies have been proposed to reduce unnecessary antibiotic use including observation, delayed prescribing, and prescribing shorter durations of antibiotics.^1^ In clinical trials, observation and delayed prescribing reduced antibiotic utilization and had similar parent satisfaction compared to immediate prescribing.^4^ However, these strategies are rarely employed in clinical practice.^3,5,6^ Furthermore, though caregiver satisfaction is a cited as a key driver of prescribing,^7^ caregiver preferences for management of AOM are poorly understood. We evaluated caregivers’ understanding of AOM and antibiotic-associated risks, preferences for management, and behavior if prescribed a delayed antibiotic.

## Methods

This study was conducted at Denver Health (DHHA) in Denver, Colorado, from September to November 2020. DHHA is a large, diverse, federally qualified health center system. Caregivers, including parents or primary medical decision makers (eg, grandparents) of children aged 6 months to 12 years who had a diagnosis of AOM in the prior 2 years, who had a documented phone number and spoke English or Spanish were included.

A descriptive survey was developed that assessed caregiver knowledge, attitudes, and beliefs regarding AOM across 4 domains: factors associated with risk of AOM, potential harms associated with antibiotics, preferences for management, and predictive behavior with delayed prescribing (Supplementary Material). The survey was reviewed by a community advisory panel and 7 caregivers from diverse backgrounds prior to distribution. The final survey was developed in REDCap and was sent to eligible caregivers via text message. The survey remained open for 45 days and up to 3 reminders were sent to nonresponders. Descriptive statistics and weighted averages for relevant questions were computed.

The DHHA Quality Improvement Committee, which is authorized by the Colorado Multiple Institutional Review Board at the University of Colorado–Denver, reviewed the project and determined that it was not human subjects research because it was part of a quality improvement project.

## Results

The survey was sent to 2,447 eligible caregivers and 101 completed it (response rate, 4.1%) Similar to nonrespondents, respondents were largely female (n = 75, 91%), Hispanic/Latinx (n = 58, 71%), and had public insurance or were self-payers (n = 53, 65%) (Supplementary Material).

### Understanding of risk factors

More than half of caregivers knew that smoke exposure (secondhand, n = 58, 58%; residue, n = 54, 54%) or sleeping with a bottle (n = 56, 56%) increased the risk of AOM. More caregivers understood that breastfeeding (n = 69, 69%), receiving the influenza vaccine (n = 67, 67%) and regular childhood vaccines (n = 61, 61%) reduced the risk of AOM (Table 1).

### Perception of AOM complications and antibiotics

Most caregivers (n = 70, 84%) were moderately or very concerned that if their child was not treated with antibiotics the infection would not go away, the child would be in pain for longer (n = 72, 87%), develop hearing loss (n = 73, 88%), or have permanent damage to the ear (n = 73, 88%). In contrast, caregivers were more likely to be moderately or very concerned about risk of future infections that are more difficult to treat (n = 56, 66%) or allergic reaction (n = 51, 59%) than side effects (n = 40, 47%) associated with antibiotics.

### Preferences for AOM Management

Caregivers were more likely to be moderately or very satisfied with an immediate antibiotic (n = 73, 82%) than a delayed antibiotic prescription (n = 62, 69%). More than half of caregivers stated they would be very unsatisfied or only a little bit satisfied with observation with in-person (n = 57, 64%) or phone follow up (n = 52, 58%) if needed. Most caregivers 66 (77%) indicated they would fill a delayed antibiotic prescription immediately; 47 (55%) would fill and give the antibiotic immediately and 19 (22%) would fill the antibiotic but only give it if needed. Caregivers did not have strong preferences for duration of therapy. In total, 62 (70%) would prefer whatever length was recommended by the doctor and 14 (16%) wanted the shortest duration needed to treat the infection. More than 90% of caregivers felt a pain management plan was important (Table 2).

## Discussion

Similar to prior studies, caregivers were highly concerned about potential complications and pain from untreated or delayed treatment and had misconceptions about the need for antibiotics.^8,9^ In contrast, caregivers were less concerned about risks associated with antibiotics. Caregivers expressed strong preferences for immediate antibiotics and most caregivers stated they would immediately fill prescriptions written as delayed. Conversely, caregivers did not have a strong preference for antibiotic duration; most wanted whichever length was recommended by their provider. Parental pressure is often cited as a key driver of antibiotic prescribing,^7^ and education for caregivers on the risks and benefits of antibiotic treatment for AOM is likely needed to reduce misconceptions and curtail prescribing.

In contrast to prior clinical trials, caregivers in a community health center environment had low satisfaction with observation and delayed prescribing and were very likely to immediately fill prescriptions written as delayed. Pragmatic studies that broadly examine antibiotic use and satisfaction with observation or delayed prescribing in typical clinical practice environments are needed. Similarly, we previously reported that providers were less likely to use delayed prescribing or observation than immediate prescribing.^3^ Further studies are warranted to assure that national approaches to AOM management and antibiotic stewardship align with the needs of caregivers and willingness of providers to follow them.

Comparatively, caregivers did not have strong preferences for duration of therapy. We previously found that providers preferred to prescribe the shortest duration of antibiotics needed.^3^ Shorter durations of antibiotics have been shown to be equally effective for most children aged ≥2 years with AOM and are associated with reduced adverse drug events.^10^ Thus, prescribing a shorter duration of antibiotics for children aged ≥2 years is likely to substantially reduce overall antibiotic exposure in a way that is acceptable to caregivers and providers.

The strengths of this evaluation include responses from a diverse population in a community-based health system. This evaluation also has limitations. Because this was a single-center project, the data are not generalizable to other organizations and evaluation of patients from diverse settings is warranted. Selection bias likely influenced the results; respondents were more likely to identify as a race other than White or Black and were more likely to have commercial insurance than nonrespondents. In addition, the response rate was low and caregivers who felt strongly about AOM management may have been more likely to respond to the survey. The low response rate may be attributed to the characteristics of our diverse patient population including technology barriers, recruitment using text messages, concerns about being identified, literacy barriers, mistrust of the medical establishment, or lack of an incentive to respond.

In conclusion, careful evaluations of caregivers’ preferences for management and providers’ willingness to adopt recommendations across diverse practice settings and populations are needed to guide AOM management and antibiotic stewardship recommendations. In particular, studies to better understand antibiotic utilization and satisfaction with observation and delayed prescribing in typical clinical practice would be beneficial. In the interim, optimizing the duration of antibiotics prescribed is likely to be a pragmatic mechanism to reduce antibiotic use in a way that is aligned with caregiver preferences.

## Supplementary Material

Supplemental Materials

## Figures and Tables

**Table 1:** Caregivers understanding of ear infections and perceived complication risks associated with treating and not treating with antibiotics (all results are represented as n (%) unless otherwise noted).

	Reduces the risk a lot	Reduces the risk a little	Does not change the risk	Increases the risk a little	Increases the risk a lot

**How much do you think the following factors change your child’s risk of developing an ear infection?**					
Exposure to secondhand smoke (*n=101*)	13 (13)	6 (6)	24 (24)	23 (23)	35 (35)
Exposure to smoke residue on clothing or hair (*n=100*)	12 (12)	7 (7)	27 (27)	30 (30)	24 (24)
Sleeping with a bottle at night (*n=100*)	8 (8)	5 (5)	31 (31)	24 (24)	32 (32)
Receiving standard vaccines recommended for children under age 2 (*n=100*)	48 (48)	13 (13)	34 (34)	4 (4)	1 (1)
Receiving an annual influenza vaccine (*n=100*)	53 (53)	14 (14)	30 (30)	2 (2)	1 (1)
Breastfeeding before child is one year old (*n=101*)	58 (57)	12 (12)	29 (29)	2 (2)	0 (0)

	Not concerned (1)	A little bit concerned (2)	Moderately Concerned (3)	Very Concerned (4)	Weighted Score (max=4)

**If your child’s ear infection is not treated with an antibiotic, how concerned are you that any of the following might occur?**					
The ear infection will not go away (*n=83*)	5 (6)	8 (10)	11 (13)	59 (71)	3.5
My child will be in pain for longer (n=83)	5 (6)	6 (7)	13 (16)	59 (71)	3.5
The ear infection will get worse (*n=83*)	5 (6)	2 (2)	11 (13)	65 (78)	3.6
The ear infection may cause hearing loss (*n=83*)	6 (7)	4 (5)	9 (11)	64 (77)	3.6
The ear infection may cause permanent damage to the ear (*n=83*)	6 (7)	4 (5)	9 (11)	64(77)	3.6
My child will miss several days of school or daycare (n=83)	16 (19)	9 (11)	19 (23)	39 (47)	3.0
I will miss several days of work, school, or other engagements (*n=82*)	24 (29)	11 (13)	17 (21)	30 (37)	2.6
**If your child takes an antibiotic for an ear infection, how concerned are you that any of the following might occur?**					
A side effect of the antibiotic (like diarrhea or rash) (*n=85*)	16 (19)	29 (34)	24 (28)	16 (19)	2.5
An allergic reaction to the antibiotic (*n=86*)	15 (17)	20 (23)	20 (23)	31 (36)	2.8
Future ear infections that will be more difficult to treat (*n=85*)	14 (16)	15 (18)	13 (15)	43 (51)	3.0

**Table 2:** Caregiver preferences for management of acute otitis media (all results are expressed as n (%) unless otherwise noted)

	Very unsatisfied (1)	A little bit satisfied (2)	Moderately satisfied (3)	Very Satisfied (4)	Weighted Score (Max=4)

**How satisfied or dissatisfied would you be with each of the following treatment options for an ear infection?**					
Prescribed an antibiotic to be taken right away (*n=89*)	10 (11)	6 (7)	16 (18)	57 (64)	3.3
Prescribed an antibiotic to take only if your child feels worse or does not improve within 72 hours (*n=89*)	17 (19)	10 (11)	20 (22)	42 (47)	3.0
Pain management without an antibiotic and a plan to follow up with the doctor ***over the phone*** if your child feels worse or does not improve within 72 hours (*n=89*)	35 (39)	22 (25)	16 (18)	16 (18)	2.1
Pain management without an antibiotic and a plan to follow up with the doctor ***in the clinic*** if your child feels worse or does not improve within 72 hours (*n=89*)	34 (38)	18 (20)	19 (21)	18 (20)	2.2

	Not important (1)	A little bit important (2)	Moderately important (3)	Very important (4)	Weighted Score (Max= 4)

**How important is it to you to have a method of pain control, like Ibuprofen (Motrin/Advil) or Acetaminophen (Tylenol), included in the treatment plan for your child’s ear infection?** (*n=89*)	3 (3)	5 (6)	18 (20)	63 (71)	3.6

	5 days	7 days	10 days	Whichever Length is Recommended by the Doctor	The Shortest Course Needed to Treat the Ear Infection

**If your child is prescribed an antibiotic for an ear infection, which antibiotic length would you be satisfied with?** (Check all that apply, *n=89*)	10 (11)	18 (20)	10 (11)	62 (70)	14 (16)

	Fill the antibiotic prescription and start the antibiotic prescription right away	Fill the antibiotic prescription right away, but only start it if your child worsens or doesn’t improve within 72 hours	Only fill and start the antibiotic prescription if your child worsens or doesn’t improve within 72 hours	Not fill the antibiotic prescription or give it to my child even if they worsen or don’t improve within 72 hours

**If your doctor provides a prescription for an antibiotic to take only if your child feels worse or doesn’t improve within 72 hours, which would you be most likely to do?** (*n=85*)	47 (55)	19 (22)	14 (16)	4 (6)

## References

[1] Lieberthal AS, Carroll AE, Chonmaitree T, The diagnosis and management of acute otitis media. Pediatrics 2013;131:e964–e999. 23439909 10.1542/peds.2012-3488

[2] Venekamp RP, Sanders SL, Glasziou PP, Del Mar CB, Rovers MM. Antibiotics for acute otitis media in children. Cochrane Database Syst Rev 2015:CD000219. 11034677 10.1002/14651858.CD000219

[3] Frost HM, Lou Y, Keith A, Byars A, Jenkins TC. Increasing guideline-concordant durations of antibiotic therapy for acute otitis media. J Pediatr 2021. doi: 10.1016/j.jpeds.2021.07.016. PMC8792957 34274307

[4] Spurling GK, Del Mar CB, Dooley L, Foxlee R, Farley R. Delayed antibiotic prescriptions for respiratory infections. Cochrane Database Syst Rev 2017;9:CD004417. 28881007 10.1002/14651858.CD004417.pub5 PMC6372405

[5] Frost HM, Monti JD, Andersen LM, Improving delayed antibiotic prescribing for acute otitis media. Pediatrics 2021;147. 10.1542/peds.2020-026062 PMC8168601 33980695

[6] Norlin C, Fleming-Dutra K, Mapp J, A learning collaborative to improve antibiotic prescribing in primary care pediatric practices. Clin Pediatr (Phila) 2021;60:230–240. 33764189 10.1177/00099228211001623 PMC9357460

[7] Mangione-Smith R, McGlynn EA, Elliott MN, Krogstad P, Brook RH. The relationship between perceived parental expectations and pediatrician antimicrobial prescribing behavior. Pediatrics 1999;103:711–718. 10103291 10.1542/peds.103.4.711

[8] Kautz-Freimuth S, Redaèlli M, Samel C, Civello D, Altin SV, Stock S. Parental views on acute otitis media (AOM) and its therapy in children—results of an exploratory survey in German childcare facilities. BMC Pediatr 2015;15:199. 26620979 10.1186/s12887-015-0516-3 PMC4666068

[9] Hansen MP, Howlett J, Del Mar C, Hoffmann TC. Parents’ beliefs and knowledge about the management of acute otitis media: a qualitative study. BMC Fam Pract 2015;16:82. 26148678 10.1186/s12875-015-0297-7 PMC4494645

[10] Kozyrskyj A, Klassen TP, Moffatt M, Harvey K. Short-course antibiotics for acute otitis media. Cochrane Database Syst Rev 2010:CD001095. 20824827 10.1002/14651858.CD001095.pub2 PMC7052812

