# Supplementary material for: Caregiver perspectives and preferences for acute otitis media management
Source: Antimicrob Steward Healthc Epidemiol. 2021 Dec 22;1(1):e69. doi: 10.1017/ash.2021.242 (PMC8950091; doi:10.1017/ash.2021.242)
Supplement: Supplementary file 1 [file S2732494X21002424sup001.docx]

**Caregivers’ perspectives and preferences for acute otitis media management**

Holly M. Frost, MD^1,2, 3^, Amy Keith, MPH^2^, Thresia Sebastian, MD, MPH^1,3^, Timothy C. Jenkins, MD ^4,5^

**Supplemental Materials: Example Survey**

**Introduction:** You have been identified as a caregiver of a child or children who were treated for an ear infection at Denver Health. We are asking you to complete this 10-minute survey so we can improve the care we provide for children with ear infections at Denver Health. There are no right or wrong answers. Participation in the survey is optional, but we greatly value your input. Your responses will be kept private.

**Understanding of AOM**

1. How much do you think the following factors change your child’s risk of developing an ear infection?

Reduces the risk a lot

Reduces the risk a little

Increases the risk a little

Does not change the risk

Increases the risk a lot

- 1. Exposure to secondhand smoke
  2. Exposure to smoke residue on clothing or hair
  3. Sleeping with a bottle at night
  4. Receiving standard recommended vaccines for children under 2 years of age
  5. Receiving an annual influenza vaccine
  6. Breastfeeding before 1 year of age

1. How satisfied or dissatisfied would you be with each of the following treatment methods for an ear infection? Please rate each method on the scales below.

Moderately Satisfied

Very satisfied

Unsatisfied

A little bit satisfied

- 1. Prescribed an antibiotic to take right away
  2. Prescribed an antibiotic to take only if your child feels worse or does not improve within 72 hours
  3. Pain management without an antibiotic and a plan to follow up with the doctor over the phone if your child feels worse or does not improve within 72 hours
  4. Pain management without an antibiotic and a plan to follow up with the doctor in the clinic if your child feels worse or does not improve within 72 hours

1. How important is it to you to have a method of pain control, like Ibuprofen (Motrin/Advil) or Acetaminophen (Tylenol), included in the treatment plan for your child’s ear infection?

Moderately Important

A little bit important

Very important

Not important

1. If your child is prescribed an antibiotic for an ear infection, which antibiotic **length** would you be satisfied with? (check all that apply)
   1. 5 days
   2. 7 days
   3. 10 days
   4. Whichever length is recommended by the doctor
   5. The shortest course needed to treat the ear infection
2. If your child takes an antibiotic for an ear infection, how concerned are you that any of the following might occur?

Moderately concerned

Not concerned

A little concerned

Very concerned

- 1. A side effect from the antibiotic (like diarrhea or a rash)
  2. An allergic reaction to the antibiotic
  3. Future ear infections that will be more difficult to treat

1. If your doctor provides a prescription for an antibiotic to take if your child feels worse or doesn’t improve within 72 hours, which would you be **most** likely to do:
   1. Fill the antibiotic prescription and start the antibiotic right away
   2. Fill the antibiotic prescription right away, but only start it if your child worsened or didn’t improve within 72 hours
   3. Only fill the antibiotic prescription and start it if your child worsened or didn’t improve within 72 hours.
   4. **Not** fill the antibiotic prescription or give it to your child even if they worsened or didn’t improve within 72 hours
2. If your child’s ear infection is **not** treated with an antibiotic, how concerned are you that any of the following might occur?

Very concerned

Moderately concerned

A little concerned

Not concerned

At

- 1. The ear infection will not go away
  2. My child will be in pain for longer
  3. The ear infection will get worse
  4. The ear infection may cause hearing loss
  5. The ear infection may cause permanent damage to the ear
  6. My child will miss several days of school or daycare
  7. I will miss several days of work, school, or other engagements

***Most ear infections in children will go away without treating with antibiotics. Severe medical problems from not treating ear infections with antibiotics are very rare.***

1. Is there anything else you’d like to share with us about your experiences with ear infections?

**Demographic Information**

1. How old are you? ___ years
2. How many children do you have? ____
3. Are you:
   1. Male
   2. Female
   3. Male-to-Female/transgender
   4. Female-to-Male/transgender
   5. Non-binary
   6. Other
4. Do you consider yourself Hispanic or Latino/a?
   1. Yes
   2. No
5. What is your race? (Check all that apply)
   1. American Indian/Alaskan Native
   2. Asian
   3. Black/African American
   4. Native Hawaiian/Pacific Islander
   5. White
   6. Other
   7. Prefer not to answer
6. What is the highest education level you have completed?
   1. Have not completed high school
   2. High school graduate, GED or equivalent
   3. Some college
   4. College degree or higher
7. What language do you speak most often at home?
   1. English
   2. Spanish
   3. Other
8. What kind of health insurance do you have now? (Check all that apply)
   1. Medicaid
   2. DFAP
   3. CHP+
   4. Private or commercial insurance
   5. None
   6. Other (describe):
   7. Don’t know

Thank you for taking this survey!

**Supplemental Table: Demographic features of survey respondents vs. non-respondents**

| Feature | **Respondents**  N (%)  N= 82^a^ | **Non-Respondents^b^**  N (%)  N = 2336 |  |
| --- | --- | --- | --- |
| **Age** *(mean ± SD; years)* | 35.8 ± 8.3 | N/A^c^ |  |
| **Number of children** *(n, range)* | 2.6 (1, 8) | N/A |  |
| **Gender** |  | N/A |  |
| Male | 4 (5) |  |  |
| Female | 75 (91) |  |  |
| Other | 0 (0) |  |  |
| **Race** |  |  |  |
| White | 34 (41) | 1792 (77) |  |
| African American or Black | 4 (5) | 281 (12) |  |
| Other | 29 (35) | 263 (11) |  |
| **Ethnicity** |  |  |  |
| Hispanic/Latinx | 58 (71) | 1658 (71) |  |
| Not Hispanic/Latinx | 21 (26) | 672 (29) |  |
| Other | 0 (0) | 6 (0) |  |
| **Highest level of education completed** |  | N/A |  |
| Have not completed high school | 7 (9) |  |  |
| High school graduate, GED, or equivalent | 24 (29) |  |  |
| Some college | 18 (22) |  |  |
| College degree or higher | 28 (34) |  |  |
| Other | 0 (0) |  |  |
| **Language preference** |  |  |  |
| English | 46 (56) | 1420 (61) |  |
| Spanish  Other | 33 (40)  0 (0) | 772 (33)  144 (6) |  |
| **Insurance** |  |  |  |
| Commercial | 17 (21) | 189 (8) |  |
| Public/Self-Pay | 53 (65) | 2115 (90) |  |
| Other  **Visit Type^d^**  Emergency Department/Urgent Care  Outpatient Clinic  Pediatrics  Family Medicine  School-Based Health Center  Inpatient  Hospital Outpatient Surgery | 12 (15)  31 (38)  50 (61)  26 (52)  20 (40)  4 (8)  1 (1)  0 (0) | 32 (1)  941 (40)  1379 (59)  880 (64)  462 (34)  37 (2)  10 (0)  6 (0) |  |

^a^ Of 101 survey respondents, 82 completed the demographics portion of the survey. Some participants incompletely completed demographic questionnaire, thus percentages

may not equal 100%.

^b^ Non-respondent data from abstracted from the patient medical record rather than from surveys and, therefore, certain demographics were not available.

^c^ Not available.

^d^ Some patients may have been diagnosed with AOM in multiple clinic locations.
